# Endocrine Perspective of Cutaneous Lichen Amyloidosis: *RET*-C634 Pathogenic Variant in Multiple Endocrine Neoplasia Type 2

**DOI:** 10.3390/clinpract14060179

**Published:** 2024-10-29

**Authors:** Alexandru-Florin Florescu, Oana-Claudia Sima, Claudiu Nistor, Mihai-Lucian Ciobica, Mihai Costachescu, Mihaela Stanciu, Denisa Tanasescu, Florina Ligia Popa, Mara Carsote

**Affiliations:** 1 Endocrinology Department, “Grigore T. Popa” University of Medicine and Pharmacy, 700111 Iasi, Romania; alexandru-florin.florescu@umfiasi.ro; 2 Endocrinology Department, “Sf. Spiridon” Emergency County Clinical Hospital, 700111 Iasi, Romania; 3 PhD Doctoral School of “Carol Davila” University of Medicine and Pharmacy, 050474 Bucharest, Romania; mihaicostachescu@gmail.com; 4 Department 4–Cardio-Thoracic Pathology, Thoracic Surgery II Discipline, “Carol Davila” University of Medicine and Pharmacy, 050474 Bucharest, Romania; 5 Thoracic Surgery Department, “Dr. Carol Davila” Central Emergency University Military Hospital, 010825 Bucharest, Romania; 6 Department of Internal Medicine and Gastroenterology, “Carol Davila” University of Medicine and Pharmacy, 020021 Bucharest, Romania; lucian.ciobica@umfcd.ro; 7 Department of Internal Medicine I and Rheumatology, “Dr. Carol Davila” Central Military University Emergency Hospital, 010825 Bucharest, Romania; 8 Department of Endocrinology, Faculty of Medicine, “Lucian Blaga” University of Sibiu, 550024 Sibiu, Romania; mihaela.stanciu@ulbsibiu.ro; 9 Medical Clinical Department, Faculty of Medicine, “Lucian Blaga” University of Sibiu, 550169 Sibiu, Romania; denisa.tanasescu@ulbsibiu.ro; 10 Department of Physical Medicine and Rehabilitation, Faculty of Medicine, “Lucian Blaga” University of Sibiu, 550024 Sibiu, Romania; florina-ligia.popa@ulbsibiu.ro; 11 Department of Endocrinology, “Carol Davila” University of Medicine and Pharmacy, 020021 Bucharest, Romania; carsote_m@hotmail.com; 12 Department of Clinical Endocrinology V, “C.I. Parhon” National Institute of Endocrinology, 011863 Bucharest, Romania

**Keywords:** skin, lichen, thyroid cancer, surgery, amyloidosis, multiple endocrine neoplasia, medullary thyroid carcinoma, *RET* gene

## Abstract

Background: Medullary thyroid carcinoma (MTC), the third most frequent histological type of thyroid malignancy, may be found isolated or as part of multiple endocrine neoplasia type 2 (MEN2). One particular subtype of this autosomal dominant-transmitted syndrome includes an association with cutaneous lichen amyloidosis, although, generally, a tide genotype–phenotype correlation is described in patients who carry *RET* proto-oncogene pathogenic variants. Methods: Our objective was to provide an endocrine perspective of a case series diagnosed with *RET*-positive familial MTC associated with cutaneous primary lichen amyloidosis amid the confirmation of MEN2. Six members of the same family had cutaneous lesion with different features (from hyperpigmented, velvety to red/pink appearance) and four of them harbored a *RET* pathogenic variant at 634 codon (exon 11): c.1900T>G, p.634G (TGC634CGC). Results: All six patients were females with the lesion at the interscapular region. Except for two women, four of these subjects were investigated and had MTC (three of them with postoperatory confirmation). The youngest affected individual was 6 years old. The three adult females were confirmed with *RET* pathogenic variant during their 30s, while the girl underwent the familial screening as a newborn. None of them had primary hyperparathyroidism until the present time, except for one subject, and two out of the three adults also had bilateral pheochromocytoma. Notably, all patients were rather asymptomatic from the endocrine perspective at the moment when endocrine tumor/cancer was confirmed, and the skin was progressively affected a few years before the actual MEN2 confirmation. Conclusions: This case series highlights the following key message: awareness of the dermatologic findings in MTC/MEN2 patients is essential since lesions such as cutaneous lichen amyloidosis might represent the skin signature of the endocrine condition even before the actual endocrine manifestations. These data add to the limited published reports with respect to this particular presentation, noting the fact that *RET*-C634 is the most frequent pathogenic variant in MEN2-associated lichen amyloidosis; females are more often affected; the interscapular region is the preferred site; the age of diagnosis might be within the third decade of life, while we reported one of the youngest patients with the lesion. The same *RET* pathogenic variant is not associated with the same dermatologic features as shown in the vignette. The same *RET* mutation does not mean that all family members will present the same skin anomaly.

## 1. Introduction

Medullary thyroid carcinoma, the third most frequent histological type among various thyroid malignancies, may be found isolated or part of the multiple endocrine neoplasia type 2 (Sipple syndrome), also including pheochromocytoma and parathyroid tumor-related primary hyperparathyroidism [1,2,3]. One particular subcategory of this autosomal dominant-transmitted syndrome includes the association with (cutaneous) primary lichen amyloidosis; while generally a genotype–phenotype correlation is described in patients who carry *RET* proto-oncogene pathogenic variants [4,5,6].

On the other hand, cutaneous amyloidosis (also named “localized cutaneous amyloidosis”) belongs to the large chapter of the amyloidosis-related conditions that are caused by the extracellular pathologic deposits of the amyloid protein (fibrils aggregates) in prior normal skin. The term “primary” applies for the dermatologic lesions (at papillary dermis) that do not have amyloidosis counterparts at the level of different nonskin sites [7,8,9]. There are four types of keratinocyte-derivate cutaneous amyloidosis: lichen, macular, nodular, and biphasic. Approximately, one out of ten patients has a familial pattern [7]. Despite previous data suggesting a male predominance, recent findings highlighted that females are more often affected [7,8]. Epidemiologic analyses showed that lichen and macular types are the more frequent, while Asian population (a prevalence of 0.98/10,000 people was reported) is more prone than Caucasians (whereas this ailment remains extremely rare) [8,9]. Typically, the patients are adults that are confirmed with the primary cutaneous amyloidosis within the fourth to sixth decade of life, while the most affected site is the interscapular region according to most authors (but not all) [8].

No specific guideline in the field of primary cutaneous amyloidosis has been released yet. Recently, dermoscopic exam proved to be an alternative noninvasive tool for the diagnosis (other than using the biopsy-based histological exam) [8,9]. No specific therapy seems highly effective nor offers a definitive cure. Recently, laser therapy was shown to add some improvement [10] in addition to local glucocorticoids and phototherapy [7,8]. Alternatively, capsaicin gel was proposed [11] as well as different drugs that are used in various autoimmune dermatologic and rheumatologic conditions, for instance, dupilumab (if, for example, atopic dermatitis was overlapped) or upadacitinib [12,13,14,15,16,17,18]. Overall, the pathogenic mechanism, despite being multiple and complex, remains a matter of debate. Repeated external stimulation, including persistent scratching, notalgia paresthetica, metabolic conditions, and long-term subcutaneous injections such as insulin in diabetic individuals are incriminated as far as we know by now [19,20]. The connection with *RET* pathogenic variants is less understood amid the confirmation of multiple endocrine neoplasia type 2, thus the importance of addressing this topic from a practical multidisciplinary approach.

### Objective

Our purpose was to provide an endocrine perspective of a case series diagnosed with *RET*-positive familial medullary thyroid carcinoma associated with cutaneous primary lichen amyloidosis (six members of the same family had the cutaneous lesion and four of them were investigated and proved to carry the 634 codon pathogenic variant at exon 11) amid the confirmation of multiple endocrine neoplasia type 2.

## 2. Case Series: The Saga of a *RET*-Positive Family Amid the Presence of the Lichen Amyloidosis

### 2.1. Admission of the Pediatric Case

This was a 6-year-old girl who was admitted for an endocrine check-up since her mother was the index case in her family confirmed with multiple endocrine neoplasia type 2. She harbored the familial pathogenic variant of the *RET* gene: c.1900T>G, p.634G (TGC634CGC), and the current presentation was delayed due to recent COVID-19 pandemic.

On admission, the patient was in good physical health according to her biological age, except for a lesion on the posterior thorax (interscapular region) with lichen amyloidosis appearance (nonpruritic lesion) which was noticed a few months prior; otherwise, she was completely asymptomatic. On current presentation, the hormonal assays were consistent for hypercalcitoninemia as well as normal hormonal profile at the level of adrenal and parathyroid glands (Table 1).

**Figure 1:**
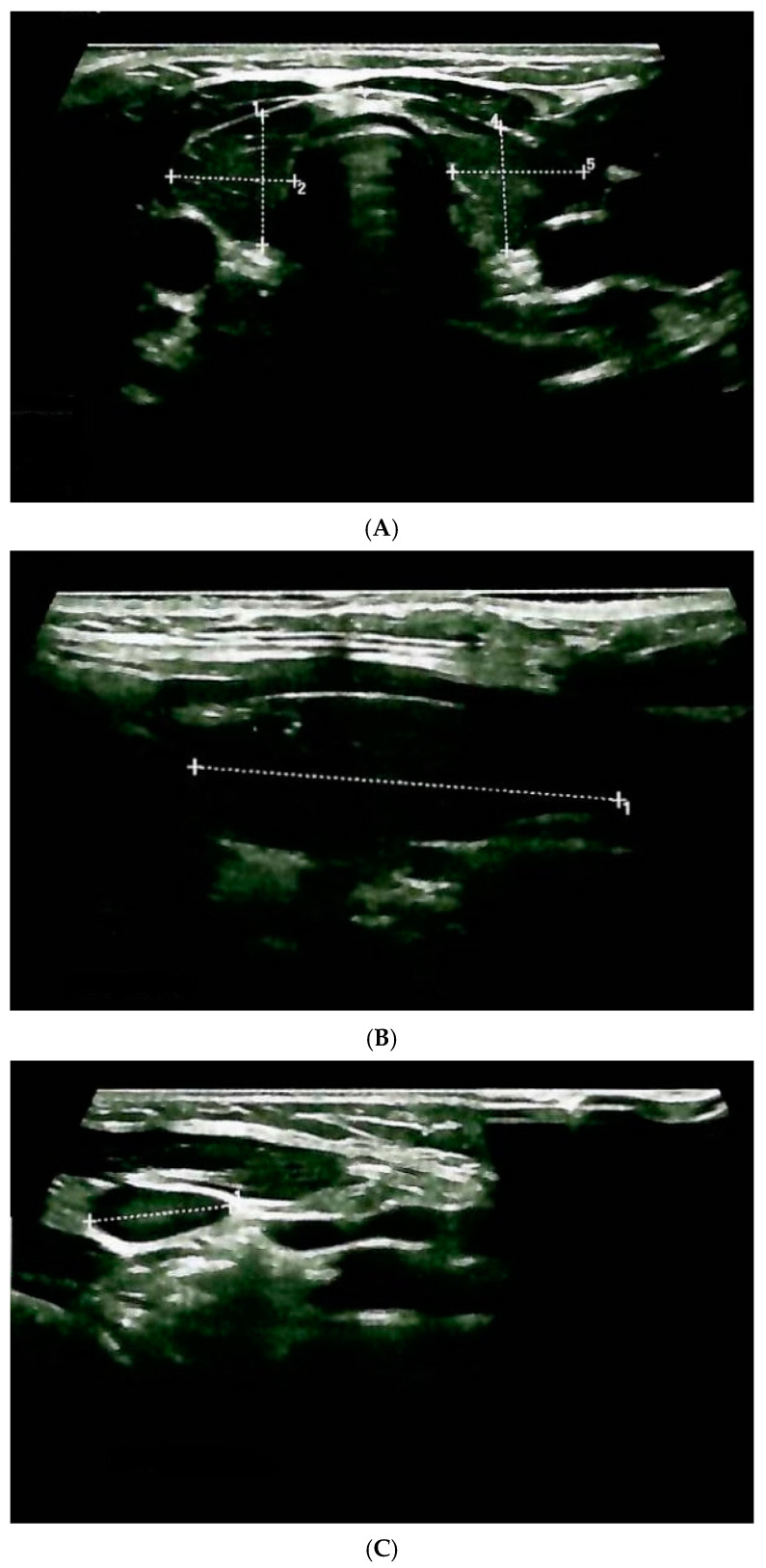

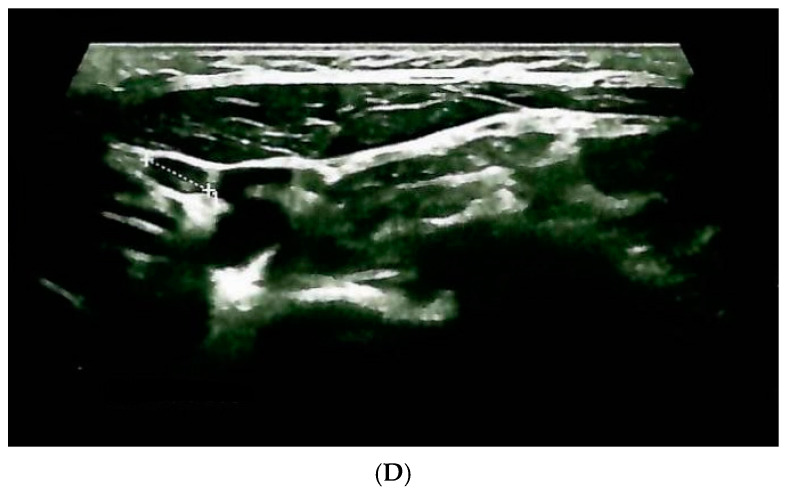
Thyroid ultrasound in a 6-year-old girl carrying *RET* pathogenic variant in codon 63. At the moment of ultrasound assessment, the patient presented high serum calcitonin levels. (**A**) Transversal plane. (**B**) Longitudinal plane of the right lobe. (**C**) Right lateral cervical adenopathy with maximum diameter of 1.1 cm. (**D**) Left lateral cervical adenopathy with nonspecific aspect and maximum diameter of 0.6 cm.

Neck ultrasound showed a right thyroid lobe of 1.1 by 1.1 by 3.2 cm and a left thyroid lobe of 1.1 by 1.1 by 2.6 cm, with intensely hypoechoic and inhomogeneous pattern (Figure 1).

Further total thyroidectomy was recommended since a medullar thyroid carcinoma was highly probable (according to her age and 634 codon involvement at exon 11 of the *RET* proto-oncogene). Additionally, post-operatory levothyroxine replacement will be mandatory, as well as life-long protocol for pheochromocytoma and primary hyperparathyroidism surveillance in addition to serial check-up of the serum calcitonin (if medullary thyroid carcinoma is confirmed).

### 2.2. Genetic Testing: Family Analysis

**Figure 2:**
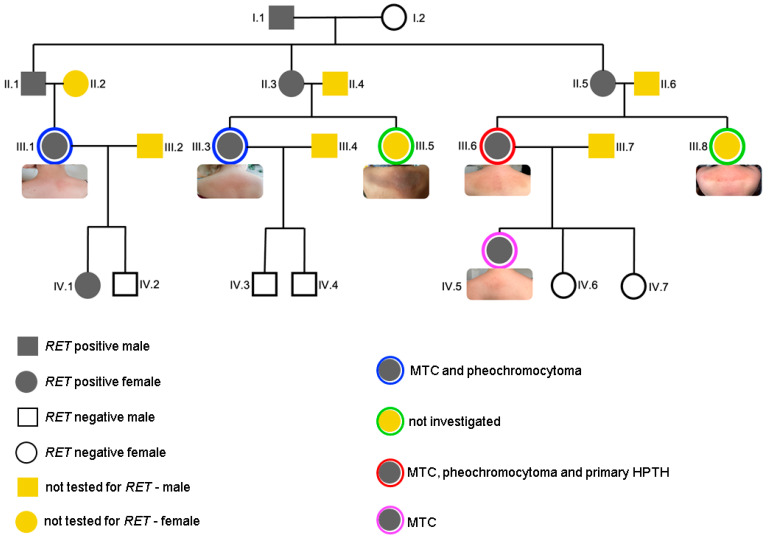
Genetic history of the *RET*-positive family with multiple endocrine neoplasia type 2 according to the available data of the family tree with regard to the genetic, endocrine, and cutaneous (lichen amyloidosis) insights (abbreviations: RET = Rearranged during transfection gene; MTC = medullary thyroid carcinoma; HPTH = primary hyperparathyroidism).

Her family has been screened for *RET* pathogenic variant, but not all members agreed for genetic testing and further endocrine assessments. Exon 11 of the *RET* gene was analyzed (located on chromosome 10q11.21) through polymerase chain reaction (PCR) and bidirectional sequencing in order to determine familial mutation c.1900T>G. The resulting sequences were compared to the Ensembl ENST00000340058. The subjects IV.5, III.6, III.2, III.1, and IV.1 were all confirmed positive, while IV.2, IV.3, IV.4, IV.6, and IV.7 were all negative. The child (patient IV.5) had two *RET*-negative siblings (IV.6 and IV.7), while their mother was the index-case (III.6) of a family confirmed with multiple endocrine neoplasia type 2. She displayed all the three endocrine components (medullary thyroid carcinoma, bilateral pheochromocytom, and primary hyperparathyroidism). The index-case (III.6) had one sister (III.8) and a female cousin (III.5) who declined any investigations until the present time (both women are in their 30s). Also, the proband has other two cousins who have been tested and found *RET*-positive: patient III.1 has two children and one of them also carried the mutation (IV.1), while the younger child was negative (IV.2), respectively, the subject III.3. has two *RET*-negative children (IV.3. and IV.4) (Figure 2).

### 2.3. Familial Lichen Amyloidosis

**Figure 3:**
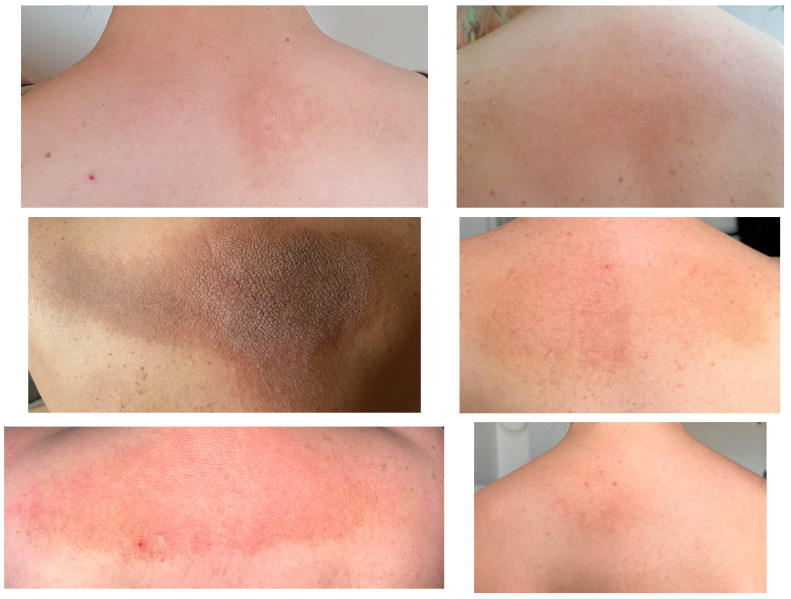
Lichen amyloidosis in a family with medullary thyroid carcinoma/multiple endocrine neoplasia type 2 (according to the family tree): patients III.1 and III.3 (**first** row on the **left** and on the **right**); III.5 and III.6 (**middle** row on the **left** and on the **right**); III.9. and IV.5 (**lowest** row on the **left** and on the **right**).

We identified six female patients with inter-scapular lesions, namely, lichen amyloidosis (patients III.1, III.3, III.5, III.6, III.9, and IV.5 according to the family tree), and only four of underwent genetic and endocrine testing (patients III.1, III.3, III.6, and IV.5). As seen in Figure 3, the most hyper-pigmented aspect was shown in patient III.5 (with a velvety plaque appearance associated with fine scales) while the most reddish appearance was in III.8, both these subjects with the largest involved area. Except for the youngest patient of 6-year-old (IV.5), all females were in their 30s and displayed the progressive cutaneous lesions a few years before. These subjects were two couples of sisters (III.3 and III.5, respectively, III.6 and III.8), and one couple was mother–daughter, as mentioned (III.6 and IV.5).

### 2.4. Overview of the Current Endocrine Status of the Other Family Members Confirmed with the Same Skin Lesion

**Figure 4:**
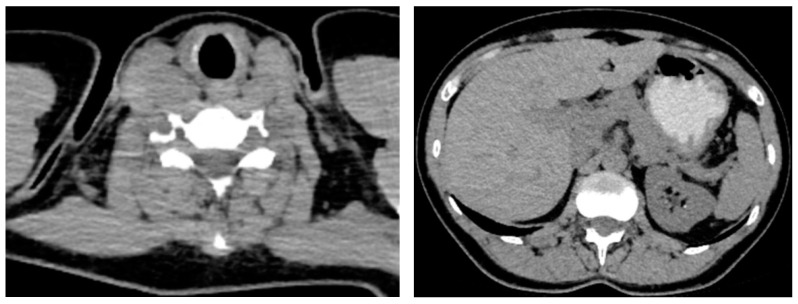
Intravenous contrast computed tomography (transversal plane): post-operatory aspect 12 years after total thyroidectomy for medullary thyroid carcinoma and bilateral adrenalectomy for bilateral pheochromocytoma: thyroid region without any aspects suggestive for remaining tissue (on the **left**); empty adrenal spaces without recurrence or remnants (on the **right**).

Patient III.6 (currently, a 43-year-old woman) was the first individual identified in her family with multiple endocrine neoplasia syndrome type 2 at the age of 31. The genetic and endocrine evaluation was followed by one-time bilateral laparoscopic adrenalectomy for pheochromocytoma (bilateral tumors of 2 cm at the level of each adrenal gland were initially detected) and a few months later she underwent synchronous total thyroidectomy, neck lymph nodes dissection, and lower left parathyroidectomy. Pre-operatory calcitonin was of 304 ng/mL and the serum total calcium was of 11.4 mg/dL with PTH (parathormone) of 122 pg/mL. She followed levothyroxine, calcium, and vitamin D, as well as glucocorticoids and mineralocorticoids replacements since the surgeries with a good clinical outcome for 12 years, including amid her pregnancy (patient IV.5) (Table 2, Figure 4).

Patient III.1 (currently, a 43-year-old woman) was tested for *RET* proto-oncogene (and identified positive) amid the diagnosis of the syndrome at her cousin (patient III.6) at the age of 30. She initially presented medullary thyroid carcinoma which was confirmed after total thyroidectomy with central neck dissection (and no lymph nodes invasion was found) with post-surgery normal calcitonin for 13 years. Two years later she was also confirmed with unilateral pheochromocytoma and underwent laparoscopic left adrenalectomy followed by a right adrenalectomy six years later for right pheochromocytoma. Following these three surgeries, she remained under levothyroxine, hydrocortisone, and fludrocortisone replacement until the present time with a good outcome (Table 3).

Patient III.3 (currently, a 44-year-old woman) was initially diagnosed with the syndrome at the age of 33 when her cousin was confirmed for the first time with the endocrine disorder (patient III.6). At that moment, she underwent synchronous surgery in terms of total thyroidectomy and central neck dissection as well as laparoscopic bilateral adrenalectomy, which confirmed the medullary thyroid carcinoma with no lymph nodes invasion and bilateral pheochromocytoma. She remained under lifelong replacement for post-operatory hypothyroidism and chronic primary adrenal insufficiency, also, along two pregnancies (both children were *RET*-negative) (Table 4).

## 3. Discussion

We introduced a visual vignette of multiple endocrine neoplasia type 2 amid the presence of the cutaneous lichen amyloidosis with different levels of severity, with all six patients being females with the lesion at the inter-scapular region. Except for two women (who declined any investigations), four of these subjects had medullary thyroid carcinoma (three of them with post-operatory confirmation). The youngest affected individual was of 6 -years -old. The three adult females were confirmed with *RET* pathogenic variant during their 30s, while the girl underwent the familial screening as a newborn. Except for one female subject, none of them had primary hyperparathyroidism until the present time, and two out of the three adults with lichen also had bilateral pheochromocytoma. Notably, all patients were rather asymptomatic from the endocrine perspective at the moment when endocrine tumor/cancer was confirmed.

**Figure 5:**
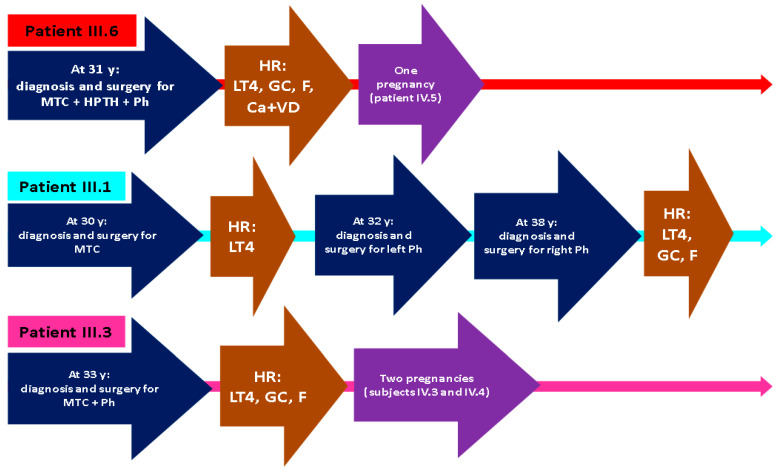
Chronology of each patient’s clinical events (abbreviations: Ca = calcium supplements; F = fludrocortisone; GC = glucocorticoids; HR = hormone replacement; LT4 = levothyroxine; MTC = medullary thyroid carcinoma; HPTH = primary hyperparathyroidism; Ph = pheochromocytoma; VD = vitamin D supplements; y = years).

The endocrine assessments in other *RET*-positive members of the family were available for the subject IV.4 who is a teenager that had already undergone total thyroidectomy associated with central neck dissection followed by the confirmation of a microcarcinoma of medullary type, and she has no skin lesion so far. Of note, all adults confirmed with lichen had the lesion a few years before the endocrine evaluation with a progressive appearance, but no specific timeline was available (Figure 5).

### 3.1. Cutaneous Lichen Amyloidosis and Daily Endocrine Practice

Lichen amyloidosis, a very rare condition, has been mostly described in relationship with chronic idiopathic pruritus (but not exclusively), and no specific therapy provides the cure. The presentation varies from hyper-pigmented aspect to red areas, papules associated with a dry skin, and clustered or desquamate-like plaques [9,21,22]. Lichen aspect comes for the deposits of amyloid, a protein that is produced by the keratinocytes [23]. By definition, no lesion other than seen at the skin level accompanies the primary cutaneous lichen amyloidosis, yet, the underlying mechanisms are unclear and some case reports of different endocrine disorders raised the question of potential common pathways. For instance, the lichen identification at the onset of iatrogenic myxedema following therapy for Graves’ disease suggested a common autoimmune involvement [24].

Another report from 2022 introduced a 55-year-old woman co-diagnosed with lichen amyloidosis and acquired reactive perforating collagenosis, while her medical records registered type 2 diabetes mellitus and a papillary thyroid carcinoma which was post-operatory confirmed [25]. Generally, skin displays an abundance of receptors to various hormones; thus, multiple endocrine and metabolic disorders are associated with challenging dermatologic expressions in regard to various genetic and epigenetic contributors [17,19,25].

### 3.2. The Cutaneous Lichen Amyloidosis Association with Multiple Endocrine Neoplasia Type 2

The most frequent pathogenic variant in *RET* proto-oncogene that has been reported in association with the cutaneous lichen amyloidosis is located at the level of codon 634 (exon 11) as described in this vignette. Approximately one third of the patients carrying this mutation will display the skin lesion across life time, typically before the endocrine conditions are recognized unless the patients are already under a screening protocol as found in our pediatric case [5,26,27,28]. However, well-designed studies (with a high level of statistical evidence) with concern to the natural history of the cutaneous lichen amyloidosis in multiple endocrine neoplasia type 2 are limited at this point.

Generally, the presence of the lichen amyloidosis is confirmed in adulthood years (within the third to the fourth decade of life) [5,29]. Early recognition might help the early identification of the endocrine conditions if the *RET* mutation is not already confirmed within one family. For example, Fang et al. [30] published in 2022 a study on 51 subjects coming from seven families with multiple endocrine neoplasia type 2 that was associated with lichen amyloidosis: females were more affected and the inter-scapular region was the most often described, while *RET*-C634 variant displayed the highest frequency, data that are consistent with our findings [30]. Moreover, Scapineli et al. [31] identified the mean age of the endocrine components of 31 ± 17 years, while the average age at the diagnosis of the skin lesion was of 20 ± 13 years [31].

Tang et al. [32] published a retrospective series in 2021 with concern to the simultaneous surgeries for patients with multiple endocrine neoplasia as seen in the three adult females across our case series. This is not an unusual scenario since screening protocols are not always accessible in many regions. Interestingly, one patient harboring *RET*-C634R pathogenic variant had, not only medullary thyroid carcinoma and pheochromocytoma, but also primary hyperparathyroidism, as seen in a single case according to our series (being the rarest component among the endocrine tumour tumor in the syndrome) [32]. Malhotra et al. [33] also described a *RET*-C634 positive female of 33-year-old who was initially diagnosed with metastatic medullary thyroid carcinoma and a left pheochromocytoma and showed a larger hyper-pigmented area of lichen amyloidosis at unilateral scapular region [33]. As mentioned, we descried an asynchronous diagnosis of pheochromocytoma at the ages of 32, respectively, of 38 in a single female subject.

**Figure 6:**
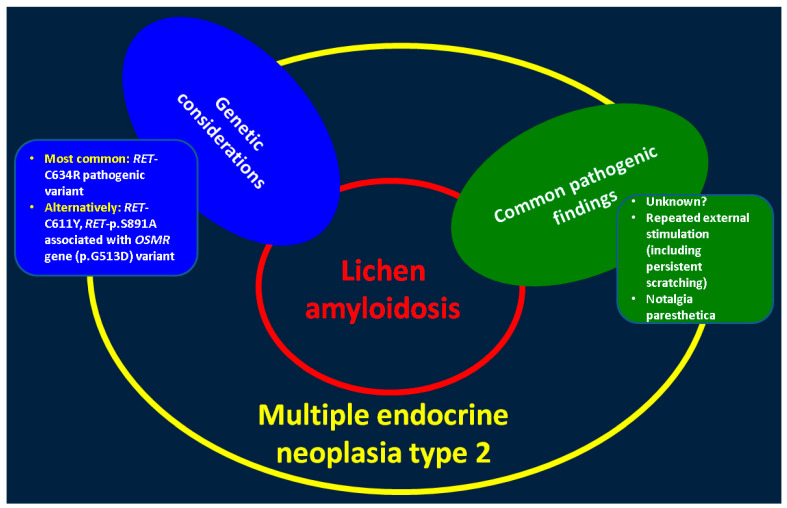
Genetic and pathogenic findings in patients with lichen amyloidosis and multiple endocrine neoplasia type 2 [19,20,34,35,36,37,38].

As exception from the C634, we highlight the study conducted by Qi et al. [34] that identified a case of germline C611Y (c.1832G>A) *RET* mutation with inter-scapular lichen amid multiple endocrine neoplasia type 2. Of note, long standing pruritus may be present years before the actual development of the lichen, but the actual cause of developing lichen in these patients remains an open issue [34]. The first *RET* p.C634F pathogenic variant with concern to this combination of endocrine and skin ailments was revealed in a Chinese patient in 2018 [35]. Alternatively, *RET*-p.S891A associated with *OSMR* gene (p.G513D) variant has been identified in lichen amyloidosis and familial medullary thyroid carcinoma [36]. The earliest diagnosis of medullary thyroid carcinoma and lichen amyloidosis was reported in 2002 in an asymptomatic 5-year-old child coming from a family with multiple endocrine neoplasia type 2 that harbored *RET*-Cys634Trp (TGC->TGG) pathogenic variant [37] (Figure 6).

### 3.3. Other Dermatologic Findings in Patients with Multiple Endocrine Neoplasia Type 2

**Figure 7:**
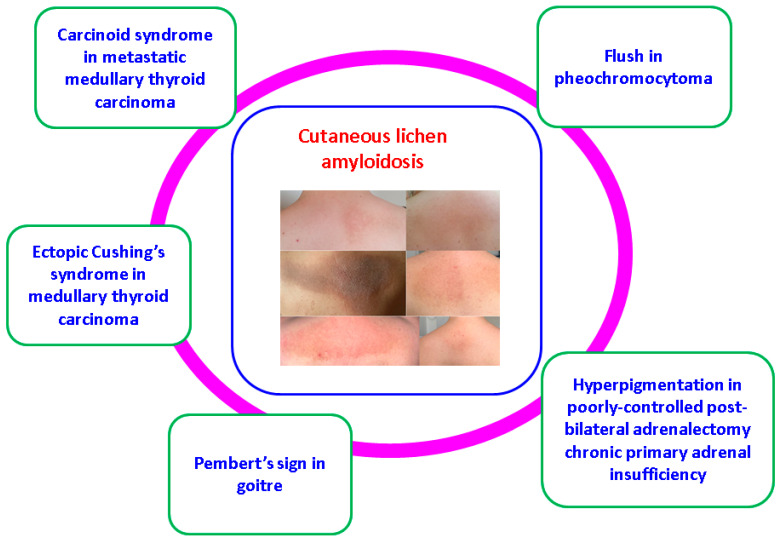
Insights of the potential skin involvement in patients diagnosed with multiple endocrine neoplasia type 2 [36,37,38,39,40,41,42,43].

On a larger perspective, a patient confirmed with multiple endocrine neoplasia type 2 might present other dermatological issues such as a carcinoid syndrome in the case of a metastatic medullary thyroid carcinoma, as seen in other neuroendocrine neoplasia with gastro-intestinal and even pulmonary origin [38,39]. Ectopic Cushing’s syndrome with typical dermatologic complications such as red stria or skin infections was rarely found in advanced medullary thyroid cancer [40]. A large, long-standing goiter (including underlying different thyroid malignancies) might cause Pembert’s sign [41,42]. Flushing has been described in sporadic or familial pheochromocytoma [43]. On the other hand, iatrogenic primary adrenal insufficiency upon bilateral adrenalectomy might cause hyperpigmentation due to high ACTH (adrenocorticotropic hormone) if the disease is incompletely or poorly controlled through medication [44,45] (Figure 7).

### 3.4. Current Limits and Further Research

Across this case series of real-life setting we provided a sample-based analysis of lichen amyloidosis within a single family harboring *RET*-C634 pathogenic variant. In addition to the fact that not all family members agreed for further assessments, no skin biopsy was provided (which is not mandatory in this particular instance, despite the fact that some authors double checked the dermatologic lesion across a histological examination [46]). Generally (regardless the presence of the endocrine ailments), in addition to the dermatologic exam, histopathological findings of skin biopsy are highly recommended to confirm the presence of the amyloid protein and to rule out other skin condition that might have a similar appearance [1,46]. Declining not only biopsy, but also the endocrine and genetic assessment by one patient represents a major aspect (limit) in real-world medicine.

Also, the impact of the family members’ refusal of the genetic testing on the overall results might not show the exact rate of association with regard to the skin involvement in this hereditary syndrome. The long term effects of untreated medullary thyroid carcinoma and pheochromocytoma are dramatic such as local and distant malignancy spreading, respectively, acute cardiovascular and neurological events (e.g., stroke, severe high blood pressure, acute myocardial infarction, etc.), diabetes or hypertension-related chronic kidney disease. In addition, the lack of testing the children of these family members might show a similar negative impact, noting there is a 50% risk of syndrome inheritance [4,5].

Across this case series we found only one patient with primary hyperparathyroidism which is the least frequent component in multiple endocrine neoplasia type 2, and the data we have so far did not confirm a stronger association with the presence of lichen amyloidosis in these patients. Parathyroid tumors have been reported in 0.1–0.3% of the population and 98% of them are benign. Genetic primary hyperparathyroidism represents 2% to 10% of all cases and germline mutations are reported in 10% of type 2A, respectively, 50% of type 2B subjects with multiple endocrine neoplasia. The parathyroid involvement is described later across life span when compare to the medullary thyroid carcinoma and the familial cases harboring *RET* pathogenic variants should follow lifelong surveillance protocols of PTH and annual calcium assays. Interestingly, *RET*-C634 represents the most common mutation associated with the identification of the parathyroid tumors, as seen in lichen amyloidosis [47,48].

On the other hand, we should mention that the surgeries’ impact on the overall quality of life should be carefully taken into consideration since lifelong levothyroxine replacement is necessary following total thyroidectomy, respectively, glucocorticoids and fludrocortisone administration after bilateral adrenalectomy for bilateral pheochromocytoma. Post-operative hypoparathyroidism, as shown above, also requires long term vitamin D and calcium supplements. So far, the studies with respect to the quality of life in this hereditary aliment are limited and the dermatologic involvement brings an additional burden to the overall picture. The aggressiveness of the medullary thyroid carcinoma remains the main contributor to the syndrome-related mortality, including in younger patients. Philological support across life span and surveillance at multidisciplinary specialized centers with regard to the specific protocols might improve the outcome [49,50,51].

Generally, the level of knowledge with regard to the lichen-related mechanisms that are connected to the *RET* proto-oncogene or to the endocrine panel of tumors/cancers remains a matter of debate and further studies are mandatory.

## 4. Conclusions

This case series highlights the following key-messages:

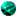
Awareness of the dermatologic findings in patients diagnosed with medullary thyroid cancer and multiple endocrine neoplasia type 2 is essential, as lesions such as cutaneous lichen amyloidosis might represent the skin signature of the endocrine condition even before the actual endocrine manifestations.
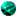
Across this real-life series we pinpointed the importance of a multidisciplinary approach.
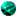
These data add to the limited published reports with respect to this particular presentation confirming the fact that *RET*-C634 is the most frequent pathogenic variant in lichen amyloidosis; females are more often affected; the interscapular region is the preferred site; the age of diagnosis might be within the third decade of life while we identified one of the youngest patients with the lesion (a 6-year-old girl).
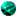
The same *RET* pathogenic variant is not associated with the same dermatologic features, as shown in the vignette.
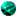
The same *RET* mutation does not mean that all family members will present the same skin anomaly.

## Figures and Tables

**Table 1:** The endocrine panel in a 6-year-old patient with lichen amyloidosis harboring *RET* pathogenic variant in a family with multiple endocrine neoplasia type 2.

Multiple Endocrine Neoplasia Type 2	Medullary Thyroid Carcinoma	Pheochromocytoma	Primary Hyperparathyroidism
Confirmation of the endocrine tumor/cancer	Highly probable	Follow-up protocol of further endocrine tumor according to the 634 codon *RET* pathogenic variant	
Age at diagnosis	6 years (*RET* genetic testing within the first year of life)		
Surgery	Recommended (planned)		
Current hormonal status	Calcitonin = **10.4** ng/mL (normal: 1–4.8)	Plasma metanephrines = 40 pg/mL (normal: 0–90) Plasma normetanephrines = 42 pg/mL (normal: 20–200)	PTH = 42 pg/mL (normal: 15–65) Total serum calcium = 9.45 mg/dL (normal: 8.4–10.2)

**Table 2:** This is the patient III.6 who was confirmed with multiple endocrine neoplasia type 2 at the age of 31 years and then she was followed for 12 years until present time (abbreviations: ACTH = adrenocorticotropic hormone; FT4 = free levothyroxine; PTH = parathormone; TSH = thyroid stimulating hormone).

Multiple Endocrine Neoplasia Type 2	Medullary Thyroid Carcinoma	Pheochromocytoma	Primary Hyperparathyroidism
Confirmation of the endocrine tumor/cancer	Yes (+2/10 cervical lymph nodes invasion)	Yes (bilateral)	Yes (left inferior parathyroid tumor)
Age at diagnosis	31 years (+ *RET* testing at 31 years)		
Hormonal assays before surgery	Calcitonin = **304** ng/mL (normal: 1–4.8)	Plasma metanephrines = **300** pg/mL (normal: 0–90) Plasma normetanephrines = **400** pg/mL (normal: 20–200)	PTH = **122** pg/mL (normal: 15–65) Total serum calcium = **11.4** mg/dL
Surgery	Yes (synchronous total thyroidectomy, neck lymph nodes dissection and selective removal of a single parathyroid tumor)	Yes (synchronous bilateral adrenalectomy)	Yes
Age at the moment of surgery	31 years		
**Current post-operatory status**			
	**Primary** **hypothyroidism**	**Chronic primary** **adrenal insufficiency**	**Hypoparathyroidism**
Latest hormonal assessment	TSH = **5.96** μIU/mL * (normal: 0.35–4.94) FT4 = 11.69 pmol/L * (normal: 9–19) * under levothyroxine 100 μg/day Calcitonin = 1.85 ng/mL (normal: 1–4.8)	Plasma metanephrines = 31 pg/mL (normal: 0–90) Plasma normetanephrines = 155 pg/mL (normal: 20–200) ACTH = 21.8 pg/mL ** (normal: 3–66) ** Under hydrocortisone 25 mg/day + fludrocortisone 0.1 mg/day	PTH = **10.76** pg/mL*** (normal: 15–65) Total serum calcium = **6.54** mg/dL *** (normal: 8.4–10.2) *** Under calcitriol 0.5 μg per day + 500–1000 mg oral calcium/day

**Table 3:** This is the patient III.1 who was confirmed with multiple endocrine neoplasia type 2 at the age of 30 years and then she was followed for 13 years until the present time (abbreviations: ACTH = adrenocorticotropic hormone; FT4 = free levothyroxine; PTH = parathormone; TSH = thyroid stimulating hormone).

Multiple Endocrine Neoplasia Type 2	Medullary Thyroid Carcinoma	Pheochromocytoma	Primary Hyperparathyroidism
Confirmation of the endocrine tumor/cancer	Yes (no lymph nodes invasion)	Yes (bilateral)	No
Age at diagnosis	30 years (+*RET* testing at 30 years)	32 years (left adrenal), respectively, 38 years (right adrenal)	No
Hormonal assays before surgery	Calcitonin = **74** ng/mL (normal: 1–4.8)	Plasma metanephrines = **148** pg/mL (normal: 0–90) Plasma normetanephrines = 78 pg/mL (normal: 20–200)	No
Surgery	Yes (total thyroidectomy + neck lymph nodes dissection)	Yes (asynchronous bilateral laparoscopic adrenalectomy)	No
Age at the moment of surgery	30	32, respectively, 38 years	No
	**Current post-operatory status**		
	**Primary** **hypothyroidism**	**Chronic primary** **adrenal insufficiency**	**Normal parathyroid status**
Latest hormonal assessment	TSH = 1.66 μIU/mL * (normal: 0.35–4.94) FT4 = 14.29 pmol/L * (normal: 9–19) * Under levothyroxine 150 μg/day Calcitonin = 1.15 ng/mL (normal: 1–4.8)	Plasma metanephrines = 15 pg/mL (normal: 0–90) Plasma normetanephrines = 23 pg/mL (normal: 20–200) ACTH = 4.38 pg/mL ** (normal: 3–66) ** Under hydrocortisone 30 mg/day + fludrocortisone 0.5 mg/day	PTH = 20.45 pg/mL (normal: 15–65) Total serum calcium = 8.9 mg/dL (normal: 8.4–10.2).

**Table 4:** This is the patient III.3 who was confirmed with multiple endocrine neoplasia type 2 at the age of 33 years and then she was followed for 11 years until the present time (abbreviations: ACTH = adrenocorticotropic hormone; FT4 = free levothyroxine; PTH = parathormone; TSH = thyroid stimulating hormone).

Multiple Endocrine Neoplasia Type 2	Medullary Thyroid Carcinoma	Pheochromocytoma	Primary Hyperparathyroidism
Confirmation of the endocrine tumor/cancer	Yes (no lymph nodes invasion)	Yes (bilateral)	No
Age at diagnosis	33 years (+*RET* testing at 30 years)	33 years	No
Hormonal assays before surgery	Calcitonin = **475** ng/mL (normal: 1–4.8)	Plasma metanephrines = **332** pg/mL (normal: 0–90) Plasma normetanephrines = **552** pg/mL (normal: 20–200)	No
Surgery	Yes (total thyroidectomy + neck lymph nodes dissection)	Yes (synchronous bilateral laparoscopic adrenalectomy)	No
Age at the moment of surgery	33 years		No
	**Current post-operatory status**		
	**Primary** **hypothyroidism**	**Chronic primary adrenal insufficiency**	**Normal parathyroid status**
Latest hormonal assessment	TSH = 1.66 μIU/mL * (normal: 0.35–4.94) FT4 = 14.29 pmol/L * (normal: 9–19) * Under levothyroxine 125 μg/day Calcitonin = 1 ng/mL (normal: 1–4.8)	Plasma metanephrines = 10 pg/mL (normal: 0–90) Plasma normetanephrines = 66 pg/mL (normal: 20–200) ACTH = 31 pg/mL ** (normal: 3–66) ** Under hydrocortisone 40 mg/day + fludrocortisone 0.5 mg/day	PTH = 52 pg/mL (normal: 15–65) Total serum calcium = 9.5 mg/dL (normal: 8.4–10.2)

## Data Availability

The research data that support the findings of this case series are not publicly available. Other medical records are available upon request in accordance with the hospital rules, patients’ consent, and local Ethics Committee.
